# Supplementary figures and images for: Molecular characterization of hepatitis B virus genotypes A and D among inmates and blood donors in Northeastern Kenya
Source: PLoS One. 2026 Feb 17;21(2):e0336726. doi: 10.1371/journal.pone.0336726 (PMC12912582; doi:10.1371/journal.pone.0336726)

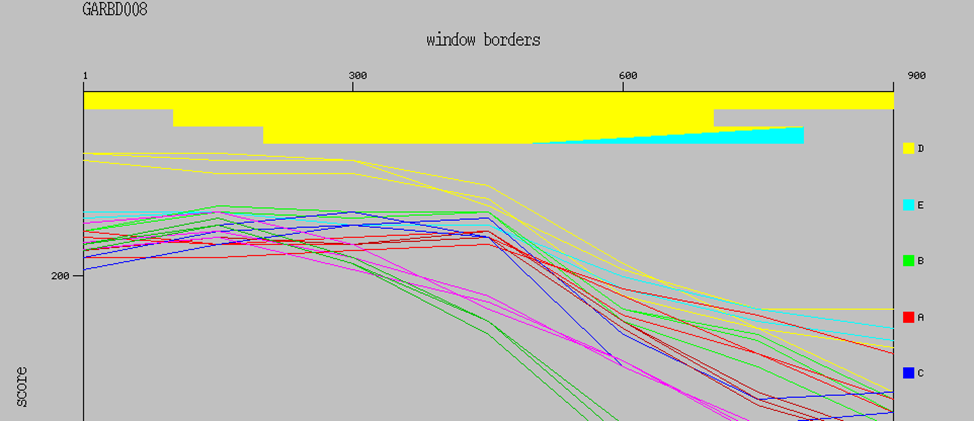

Supplement: S1 Fig — (TIF) [file pone.0336726.s001.tif]

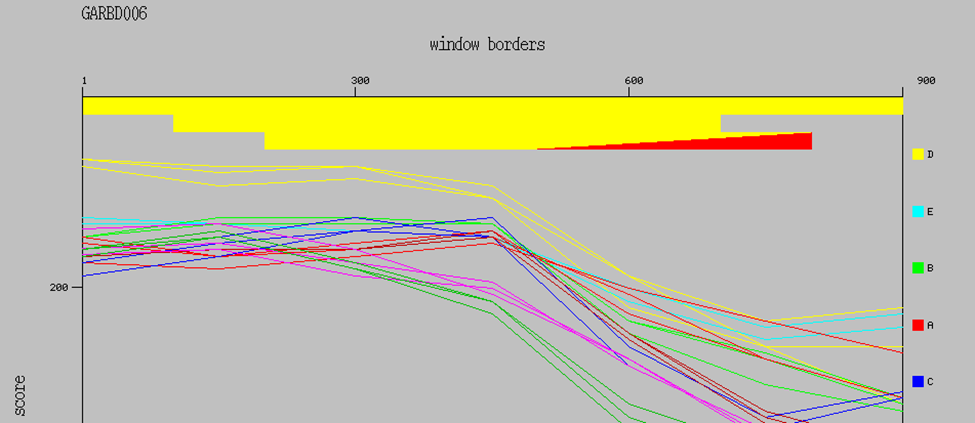

Supplement: S2 Fig — (TIF) [file pone.0336726.s002.tif]
