# Supplementary material for: Molecular characterization of hepatitis B virus genotypes A and D among inmates and blood donors in Northeastern Kenya
Source: PLoS One. 2026 Feb 17;21(2):e0336726. doi: 10.1371/journal.pone.0336726 (PMC12912582; doi:10.1371/journal.pone.0336726)
Supplement: Attachment — Submitted filename: Response to Reviewers.doc [file pone.0336726.s004.doc]

**27^th^ December 2025**

**LETTER OF REBUTTAL**

**Authors’ note to the Editor**

We sincerely thank the reviewers for taking their time to go through our manuscript and for their thorough review which we believe greatly improves the quality of our manuscript. We also appreciate their generous comments and we have revised our manuscript to address their concerns.

We wish to respond to the specific comments that required our answers/comments as follows;

**Reviewer #1**

**Abstract**The authors should proofread the abstract; it seems AI was used to generate the abstract, hence there are many typos which need to be worked on - **The authors have slightly reviewed the abstract for typos and also wish state that at no time in the writing of this manuscript was AI used.**

**Introduction**

1. Some sentences could be more concise, for example, “These genotypes have shown a propensity for intergenotypic recombination…” could be tightened for flow. - **We concur and** t**his has now been revised for better flow.**
2. The citation style (e.g., “Makhoha et al., 2023 recently published...”) should be in past tense without “recently,” as that becomes outdated; also, remove first names in scientific writing. - **We agree with the reviewer and have revised as guided.**
3. The paragraph on study populations (inmates vs. donors) is informative but slightly repetitive, it reiterates content from the methods and could be condensed. - **We are in agreement and have deleted a sentence from this paragraph without losing the intended meaning.**
4. The final sentence (“in line with the global agenda…”) is effective but would benefit from a stronger closing statement emphasizing the novelty or expected contribution of this study. - **We are in agreement and have revised the sentence for a stronger closing statement.**

**Methods**

1. Writing and organization
a) Some sentences are too long or contain redundant information, e.g., lines 135–139 could be shortened or merged for clarity. - **we have reviewed the sentence for brevity and clarity.**
b) There are minor grammatical inconsistencies (“then sequences” “then sequenced”; “was then sequences by bidirectional…” “was then sequenced bidirectionally”). Suggestion: Edit for flow and consistency (use past tense uniformly, e.g., “was performed,” “was analyzed”). - **Apologies, this has now been corrected.**
2. Scientific details missing or vague
a) Primer sequences are not explicitly listed; the reference “(17)” should be clearly identified and verified. One would expect at least the forward and reverse primer sequences or accession numbers. **- The reference (Chook et al., 2015) has now been indicated, these are universal primers commonly used in HBV molecular studies. The primer co-ordinates are also given.**
b) The annealing temperatures and cycle numbers are provided, but the amplicon size (bp) is not stated. - **the amplicon size has been added after the description of the second round PCR.**
c) The sequencing service could specify location or facility (e.g., “sequenced at Kenyatta University molecular lab or outsourced to …”). **- the sequencing facility has been identified in the last paragraph of this section.**
d) It is unclear how quality control of sequences (trimming, contamination check) was performed before alignment. Suggestion: Add brief information on amplicon size, primer details, and QC filtering steps. **- a brief description of this has been included in the revision in the data analysis section.**
e) Although the use of MrBayes is correct, the model of nucleotide substitution (e.g., GTR+G+I) used in the Bayesian analysis is not mentioned. Tree validation metrics (posterior probability thresholds, burn-in value, number of independent runs) are missing. **- this has now been added in the revision to include the substitution model, burn-in and number of runs.**
f) There is no mention of how mixed genotypes or ambiguous bases were handled. Suggestion: Include these parameters to strengthen reproducibility and perceived analytical rigor. **- this information has been added in the MrBayes parameters description.**The overall tone is informative, but the phrasing could be made more formal and concise.Example revision:“Approximately 4 mL of venous blood was collected into plain vacutainers from each participant” is preferred over “Approximately 4 ml of blood was collected from a selected vein of each individual into a plain vacutainer.” **- we agree with this comment and have revised the sentence.**

**Results**

1. Seroprevalence of HBV
a) The confidence interval (CI) or exact chi-square value, which would improve statistical transparency is not defined or included. The phrase “did not reach statistical significance (p=0.31)” is correct but should be contextualised through discussing sample size limitations or low statistical power. Table 1 is mentioned but not fully integrated into the text (no data summary within the text). - **The chi-square value has been added, pertinent data summary of Table 1 is already included in the text.**
b) The Results section omits any age, sex, or risk factor stratification, which could enrich epidemiological interpretation. The seroprevalence analysis is scientifically sound but statistically underdeveloped and somewhat descriptive. Including more analytical insight (e.g., odds ratios or subgroup trends) would strengthen the epidemiological message. **- This was intentionally left out in the current manuscript as this was included in a previous manuscript whose focus was on the seroprevalence results.**
2. HBV DNA Detection and Genotyping
a) The authors report that “several seronegative samples” were successfully amplified this intriguing observation is scientifically important but underexplained. It suggests occult HBV infection (OBI), which warrants at least a brief acknowledgement even in the Results section. - **We appreciate this comment and have acknowledged this observation.**
b) The genotype distribution table is referred to as Table 1, but it should be Table 2 to maintain consistency (Table 1 has already used for seroprevalence). **- Apologies for this, the correction has now been made.**
c) The authors might clarify how the 22 samples were selected; were these all HBsAg-positive, or did they include a random subset? This is key for interpretation. **- We appreciate this comment and have explained the selection of the 22 samples in the revision.**
3. Phylogenetic Analysis
a) The latter probability (91%) is acceptable but could be accompanied by a mention of tree topology metrics or branch support for other genotypes for completeness. **- A new sentence has now been added to report the branch support for the other genotypes.**
b) The statement “distinctly clustered away from other sub-genotypes” should be supported by describing whether this divergence indicates a new lineage, regional adaptation, or recombination. **- Since this observation contrasted with the geno2pheno results, the possibilities mentioned were considered necessitating recombination analysis. This was outlined in the discussion section.**
c) The recombination results are briefly stated without genomic detail (e.g., breakpoint positions, affected regions). **- The breakpoint positions have now been stated in the revision, additionally, the sequenced region is identified as the P/S gene.**
d) The analysis would be stronger if the authors indicated the potential epidemiological or clinical implications of these recombinants, e.g., whether such patterns are consistent with previous East African HBV diversity. **- We are in agreement and have included this in the discussion section.**

**Discussion**

The authors open by linking their study to the UN SDG 3.3 and WHO’s 2030 hepatitis elimination goals, a globally relevant framing that gives the discussion strong policy alignment. They effectively justify the study’s focus on underrepresented populations (inmates, Northern Kenya), demonstrating clear research need.

a) However, the paragraph reads slightly like an introduction rather than a discussion. While context is valuable, a more analytical transition (e.g., “Our findings contribute to this goal by…”) would make it flow better into interpretation. The Citations to WHO and SDG literature could be condensed to avoid redundancy. The contextual introduction is excellent in scope but could be streamlined for brevity and better transition into study interpretation. **- We sincerely appreciate this and are in total agreement. We have now revised this paragraph to fit the context.**The following should be worked on :
b) There is little exploration of potential behavioural or environmental factors driving higher inmate prevalence (e.g., overcrowding, shared instruments, limited healthcare access). **- We appreciate this comment and have included these factors with relevant citations in the revision.**
c) The text is descriptive and could integrate a short epidemiological interpretation beyond numeric comparison. The discussion is well-documented and comparative, but under-discussed regarding underlying causes and implications for screening policy. **- this observation is correct, however this was done to avoid an overlap with the seroprevalence paper.**
d) The text could make clearer whether genotype D dominance was observed across both populations or more pronounced in a specific one (this distinction is important for epidemiologic inference). - **we concur with the reviewer and have explained this in the revision.**
e) The discussion would benefit from hypothesising about possible sources or routes of introduction, for instance, historical or cross-border movement from Somalia or Ethiopia. **- We had included this by hypothesizing that it was “through migration or trade”**
f) A sentence or two connecting genotype D’s clinical implications (e.g., treatment response to tenofovir or interferon) would enhance translational impact. **- We agree with the reviewer that this is important and included a sentence on it in the revision.**
g) This section is scientifically rigorous and insightful but could emphasise why genotype D matters biologically beyond its novelty. **- This had been mentioned but the increased rate of escape mutations in genotype D has been added.**
h) The recombination discussion (lines 290–310) becomes somewhat repetitive, restating similar concepts (migration, cross-border mixing, recombination potential). It could be condensed without losing meaning. - **We agree with the reviewer and have greatly revised the recombination discussion for clarity.**
i) The authors could explicitly indicate whether their D/A and D/E recombinants share breakpoint regions previously reported in Africa; this would strengthen the evolutionary argument. **- We are in agreement with the reviewer and have cited several studies from Africa that have reported these recombinants in the revision.**
j) (Lines 316–340), Public Health and Diagnostic Implications were explicitly discussed; however, the potential impact on therapeutic outcomes could be supported by referencing known genotype D-associated resistance patterns (e.g., lower interferon response). **- We agree that this is an important point and have added two sentences to support the public health implications of genotype D.**
k) The final recommendations could briefly mention capacity-building needs (infrastructure, partnerships) for genomic surveillance in Kenya. **- We appreciate this comment and have revised the final recommendations accordingly.**

**Reviewer #2**

1. The mention of genotype D in the title is ambiguous, as there are also reports on genotype A. Please clarify whether genotype A samples were excluded from the analysis or adjust the title accordingly. **- Thank you for this comment, we regret this mistake. In a bid to report the unusual trend we obscured genotype A from the title. We have now revised the title accordingly.**Manuscript Text:
a) Review the manuscript for grammar, sentence structure, and scientific writing style. - **We have endeavored to review this in the revision.**
b) Correct issues related to sentence case, punctuation (commas, full stops, etc.), and overall readability. - **We have checked on this and made a few revisions to improve readability.**
c) Revise unclear or awkward sentences as previously suggested. **- Identified awkward sentences have now been revised.**
d) Remove redundant or misplaced information throughout the text to improve flow and conciseness. - **we have endeavored to generally improve the flow of the text.**
e) Include missing details on statistical analysis, including the frequency and distribution of results. -**The missing details identified by reviewer #1 have been incorporated in the revision.**
f) Avoid repetition of information, especially in the results and discussion sections. **- We are in agreement and have removed the identified repetitions pointed out by reviewer #1.**
g) Ensure all factual statements are properly referenced where applicable. **- All efforts have been made to reference he factual statements.**
h) Add missing details on reagents and materials, including manufacturer and source (e.g., ExoSAP-IT™ (ThermoFisher Scientific, CA, USA)). **- The details of the PCR reagents have been added that were erroneously missed out.**
i) The discussion section still requires improvement—strengthen the interpretation of findings, ensure logical flow, and link results to relevant literature. **- Several improvements pointed out by reviewer #1 have been incorporated in the revised draft.**

**Reviewer #3**

Below are some information required:

1. A flow diagram would be useful to assist with final number of specimens for analysis (denominator) **- The total number of samples had been included, however the number of HBV DNA samples was missing and has now been included in the revision.**
2. Demographic information of participants is lacking and important **- The demographic information was intentionally left out as it was part of a previously published manuscript.**
3. Serological testing requires further information and expansion (interpretation of positive and negative results, errors, inconclusive results) **- This has not been included due to the simplicity of the test cassette and the fact that no inconclusive / invalid results were observed.**
4. Line 134-138 requires revision and clarity for better understanding. - **We agree with this comment and have revised the two sentences for clarity.**
5. Data analysis should be separated into bioinformatic analysis and statistical analysis (software used for calculations) **- Thank for this comment, the statistical analysis bit had been left out but has now been added.**
6. line 227-228 requires revision and very general and vague **- We are in agreement and have greatly revised this paragraph for conciseness.**
7. line 238 needs revision and analyzed data **- We have indicated the data in the next sentence and hope that this is sufficient.**

We hope that the reviewed version meets your expectations.
